# Phase transformation and deformation behavior of NiTi-Nb eutectic joined NiTi wires

**DOI:** 10.1038/srep23905

**Published:** 2016-04-06

**Authors:** Liqiang Wang, Cong Wang, Lai-Chang Zhang, Liangyu Chen, Weijie Lu, Di Zhang

**Affiliations:** 1 State Key Laboratory of Metal Matrix Composites, Shanghai Jiao Tong University, Shanghai 200240, China; 2 Department of Materials Science and Engineering, Northwestern University, Evanston, Illinois, 60208, USA; 3 School of Materials and Metallurgy, Northeastern University, Shenyang, 110819, China; 4 School of Engineering, Edith Cowan University, 270 Joondalup Drive, Joondalup, Perth, WA 6027, Australia; 5 School of Mathematics and Science, Jiangsu University of Science and Technology, Zhenjiang, Jiangsu 212003, China

## Abstract

NiTi wires were brazed together *via* eutectic reaction between NiTi and Nb powder deposited at the wire contact region. Phase transformation and deformation behavior of the NiTi-Nb eutectic microstructure were investigated using transmission electron microscopy (TEM) and cyclic loading-unloading tests. Results show that R phase and B19′ martensite transformation are induced by plastic deformation. R phase transformation, which significantly contributes to superelasticity, preferentially occurs at the interfaces between NiTi and eutectic region. Round-shaped Nb-rich phase with rod-like and lamellar-type eutectics are observed in eutectic regions. These phases appear to affect the deformation behavior of the brazed NiTi-Nb region *via* five distinct stages in stress-strain curves: (I) R phase reorientation, (II) R phase transformation from parent phase, (III) elastic deformation of reoriented martensite accompanied by the plastic deformation of Nb-rich phase and lamellar NiTi-Nb eutectic, (IV) B19′ martensitic transformation, and (V) plastic deformation of the specimen.

NiTi alloys are widely used as shape memory alloys (SMAs) in biomedical fields due to their excellent shape memory effect, superelasticity, low stiffness, and good biocompatibility^1^,^2^,^3^. Thanks to the phase transformations (i.e. B2 → R/B2 → B19′) occurred in NiTi alloys which are responsible for exhibiting both shape memory effect and superelasticity. The aforementioned phase transformations depend on their composition and the thermo-mechanical treatment. Recently, Nb-containing NiTi SMAs have attracted considerable interest due to their wide transformation hysteresis and good shape memory effect. And it is important to understand the phase transformation related to the shape memory effect in TiNiNb alloy. Kim *et al.*^4^ reported that only B2 to B19′ (M) phase transformation is observed in the annealed near-equiatomic NiTi alloys. On the other hand, B2 → R phase transformation takes place in near-equiatomic NiTi alloy due to the precipitation-induced inhomogeneity in both composition and internal stress fields of matrix^4^. It has been found that either martensitic or austenitic phases are obtained in NiTi-Nb alloy for a wide range of temperature during thermo-mechanical treatments^5^. Hao *et al.*^6^ reported large elastic strain and high strengh in bulk Ni_41_Ti_39_Nb_20_ (at.%), which resulted from the stress-induced B19′ phase transformation during tensile deformation. It has been reported in Ni-Ti SMAs that all phase transformations to and from B19′ phase are isothermal and other phase transformations between B2 → R and B2 → B19 are athermal^7^. These could guide in understanding the phase transformation in TiNiNb SMAs. Based on the results in literature, there are several factors that influence the transformation from B2 matrix to R phase in TiNiNb alloy. For example, inhomogeneity in matrix causes B2 → R phase transformation^8^. The addition of alloying element, such as Fe^9^ or Al^10^ also causes the B2 → R phase transformation.

Systematic studies have been conducted on the stress-induced martensitic transformation and its reorientation process in TiNi and TiNiNb^11^,^12^,^13^. It is believed that the alteration of internal elastic energy and an increase in irreversible energy during deformation, accompanying martensite reorientation, would contribute to martensitic stabilization in near-equiatomic NiTi alloys^14^. Lin *et al.*^15^ found that dislocations and vacancies created during deformation could enhance martensitic stabilization. For Ni_47_Ti_44_Nb_9_ SMA, soft β-Nb eutectic phase formed during solidification process effectively stabilizes austenite phase by absorbing large strain energy during the formation of stress-induced martensite. Thus, the transformation temperature hysteresis of NiTi-Nb SMA widens^16^. In the early stage of plastic deformation, β-Nb phase relaxes the elastic strain energy of B2 matrix phase, thereby postponing the process of martensitic transformation^16^. Generally speaking, martensite phase transformation and its reorientation are deemed to be the key factors in improving the shape memory effect and superelasticity in NiTiNb alloys.

Regarding the preparation of NiTi-Nb alloy, Grummon *et al.* adopted reactive-brazing process to form a NiTi-Nb phase *via* bonding pure Nb foils to NiTi foils, resulting in open-cell NiTi honeycombs with excellent shape-memory properties^17^,^18^,^19^,^20^,^21^. Bansiddhi and Dunand^22^,^23^ used liquid NiTi-Nb eutectic phase to bond NiTi powder to create porous NiTi. As metallurgical bonding at contact point improves both strength and stiffness, depositing brazing paste at contact point and dipping woven structure in powder slurry are generally required during specimen preparation^24^,^25^. Soft β-Nb and NiTi-Nb eutectic in different morphologies are expected to affect the superelasticity of alloys.

According to the quasi-binary NiTi-Nb eutectic phase diagram, when NiTi and Nb contacts each other, a quasi-binary liquid eutectic phase with a chemical composition near Ni_38_Ti_36_Nb_26_ (at.%) is formed^17^,^21^,^26^. Extensive efforts have been made on investigating the microstructure and mechanical properties of the eutectic Ni_47_Ti_44_Nb_9_ (at.%) alloys^16^,^27^, including the experimental evidence of thermal induced self-accommodating martensite^27^. The mechanism of B19′ martensites and R phase transformation, especially at the interfaces of NiTi-Nb brazed regions, is highly associated with superelasticity. To our knowledge, the mechanism of B19′ martensites and R phase transformation in NiTi alloy has been well studied for many years. For example, Liu *et al.*^28^,^29^,^30^,^31^ have carried out systematic studies on the pseudoelastic behavior of NiTi alloy. However, the deformation behavior of NiTi-Nb brazed NiTi wires is not systematically studied and thus the fundamental understanding of phase transformation and superelasticity in TiNiNb SMAs remains substantially unknown.

The proportion of NiTi and Nb is very important for determining the phase composition with regard to sintering temperature and time. However, sintering time is not so important. In the present study, the content and proportion of the powder mixture were altered to tailor the microstructure and composition of the bonding, which is different from the previous literatures where such control was not applied. Based on our previous results^26^, Nb-rich phase is mostly distributed around the interface of proeutectic phase and eutectic phase. However, the amount of Nb-rich phase appears to be high when the weight ratio of NiTi and Nb is designed as 1:1 (less than the weight ratio 1.6:1 for the eutectic phase), which needs much more NiTi to form full NiTiNb eutectic. In this work, NiTi wires were brazed together *via* eutectic reaction between NiTi and Nb powder deposited in wire contact region. The mixture of NiTi and Nb powder was used to braze NiTi wires using *in situ* reaction process, focusing on the certain proportion of NiTi and Nb. The liquid eutectic formed in this work wet the wires, thus filling the gap between two adjacent wires. Phase transformation and deformation behavior of the NiTi-Nb eutectic microstructure, especially the associated superelasticity, were investigated using transmission electron microscopy and cyclic loading-unloading tests. The microstructure in eutectic region and its effect on the deformation behavior of brazed NiTi-Nb were also discussed.

## Methods

### Specimen preparation

**Figure 1:**
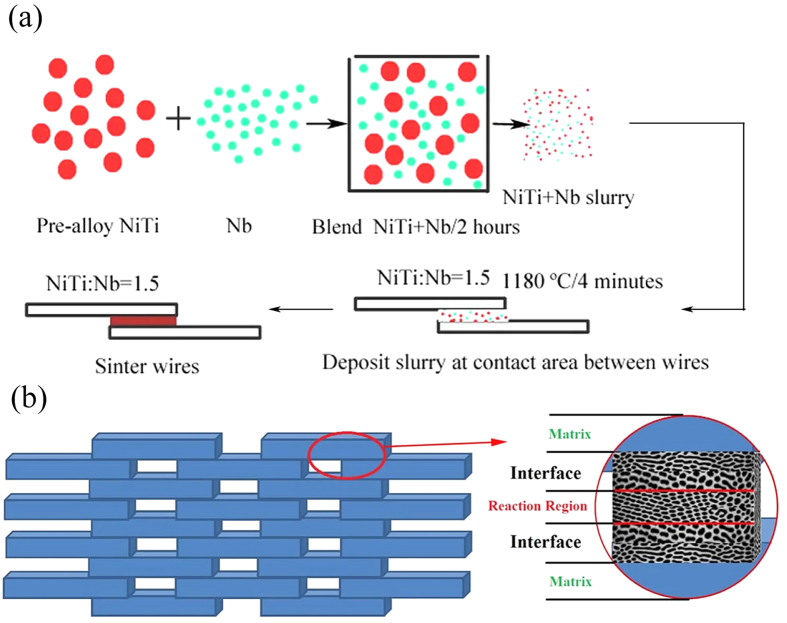
Schematic illustration for the preparation of NiTi-Nb eutectic joined parallel NiTi wires: (**a**) preparation of parallel NiTi wires with NiTi/Nb slurry added at each node, (**b**) sandwich architecture with enlarged metallurgical bonding.

Commercial-grade NiTi wires with a nominal composition of 50.5 at.% Ni-Ti and a diameter of 300 μm were obtained from Xi’an Saite Metal Materials Development (China). The ingot of commercial-grade NiTi alloy was prepared by vacuum induction melting, and then hot rolled into a roll with a diameter of 5 mm at 850 °C, followed by solid solution heat treatment at 850 °C for 1.8 ks. Then the specimen was drawn to wire with the diameter of 0.3 mm. All the samples were solution-treated at 850 °C for 1 h and water quenched to obtain homogeneous solid solution, and then aged at 450 °C for 0.5 h. The wires were polished to remove surface oxides using an automatic polishing machine outfitted with SiC paper and polishing cloth, then cut to make cylindrical shape sample having 30 mm length prior to brazing. Pre-alloyed NiTi powder (44–63 μm, 48.6 at.% Ni, Special Metals Corp., NY) and pure Nb powder (99.8% purity, 1–5 μm, Alfa Aesar, MA) together with polyvinyl alcohol PVA (87–89% hydrolyzed, high molecular weight, from Alfa Aesar, MA) were used. The NiTi wires were bonded using following six steps: (i) weight ratio of NiTi and Nb was designed as 1.5, (ii) 0.5 g NiTi and the appropriate amount of Nb powder were blended and tumbled for 2 h, (iii) the blended NiTi/Nb powder and 0.04 g PVA was added to 0.6 ml H_2_O to create a slurry, (iv) slurry at contact area between two parallel partially overlapping NiTi wires was deposited using a syringe, (v) thereafter water and PVA were removed by heating at 250 °C for 1 h in air, and (vi) NiTi/Nb powder were melt by heating to 1180 °C and kept/maintained the liquid eutectic at this temperature for 4 min to bond the wires in a high vacuum (10^−5^ torr) furnace with pure titanium getters. The sintered specimen is aged at 520 °C for 30 min followed by water quenching. Parallel brazed wires were used to analyze the superelasticity during loading and unloading cycle tensile testing. Figure 1 shows a schematic illustration of the parallel bonded NiTi wires where NiTi/Nb slurry is added at each node. Figure 1(a) shows the detailed preparation procedures of the aforementioned sandwich structure. Sandwich architecture with metallurgical bonding of reaction region and interface was achieved (Fig. 1(b)).

### Tensile testing

Tensile properties were evaluated through cyclic loading–unloading tensile testing. In order to compare, three different groups of NiTi(Nb) specimens, i.e. unbrazed NiTi wires, tensile specimen including only brazed area, and tensile specimen including both brazed area and pure NiTi wires, were used to obtain superelastic properties. Prior to tensile testing, pure NiTi wires were treated at 1180 °C followed by aging at 520 °C. Tensile tests were carried out at a strain rate of 1.0 × 10^−4^ s^−1^ at room temperature. The gauge length and the diameter of the tensile specimen were 15 mm and 0.3 mm, respectively for the pure NiTi wires. For the tensile specimen including only brazed area, the gauge length and the cross-sectional area were 4.5 mm (only brazed area) and 0.28 mm^2^. For the tensile specimen including both brazed area and pure NiTi wires, the gauge length and the diameter of the tensile specimen were 15 mm and 0.3 mm respectively, including brazed area (6.2 mm in length). It is well known that martensitic phase transformation associated with plastic deformation mechanisms could be revealed by the cyclic loading–unloading curves. In the first cycle, specimen was loaded to a displacement of 0.23 mm and then unloaded. The maximum tensile displacements during cycling testing were increased to 0.37, 0.6, 0.9, 1.2, 1.5 and 1.8 mm, respectively.

### Microstructure characterization

A Hitachi SU8030 Scanning electron microscope (SEM) at an acceleration voltage of 15 kV was used to observe the microstructure. Martensite phase transformation, dislocations and phase morphology of eutectic were observed using transmission electron microscopy (TEM). A JEOL JEM 2100 TEM equipped with EDS operated at 200 kV was used. The brazed areas including both NiTi matrix and eutectic region were investigated using TEM.

## Results and Discussions

### Microstructural characterization

**Figure 2:**
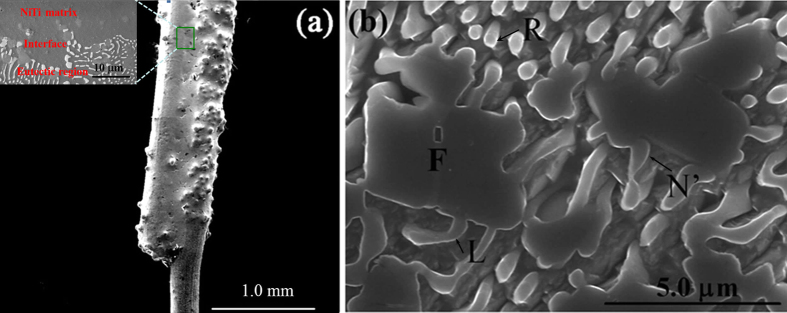
SEM micrographs of (**a**) the bonded parallel NiTi wires and (**b**) eutectic region in the polished brazed region. R and L are rod-like and lamellar-type eutectic microstructure respectively; N’ is rounded Nb-rich phase; F is facetted Ti-rich particle.

Two parallel wires were used to produce NiTi/Nb eutectic at about 1180 °C (above the eutectic temperature of 1170 °C in the NiTi-Nb quasi-binary phase diagram^17^). Figure 2(a) shows the SEM image of the as-sintered specimen. Transverse section of the brazed wire consisting of NiTi matrix, interface and eutectic region is also observed. Figure 2(b) displays the microstructure of the brazed region. It is clear that round-shaped Nb-rich phases (N’), rod-like (R) and lamellar-type (L) eutectic microstructure are observed (Fig. 2b). In addition, facet Ti-rich particles are also present (marked by F). It is reported that rod-type eutectic is obtained when the volume fraction of Nb phase in NiTi-Nb eutectic is below a critical volume (about 30%) for lamellar-type eutectic^12^.

**Figure 3:**
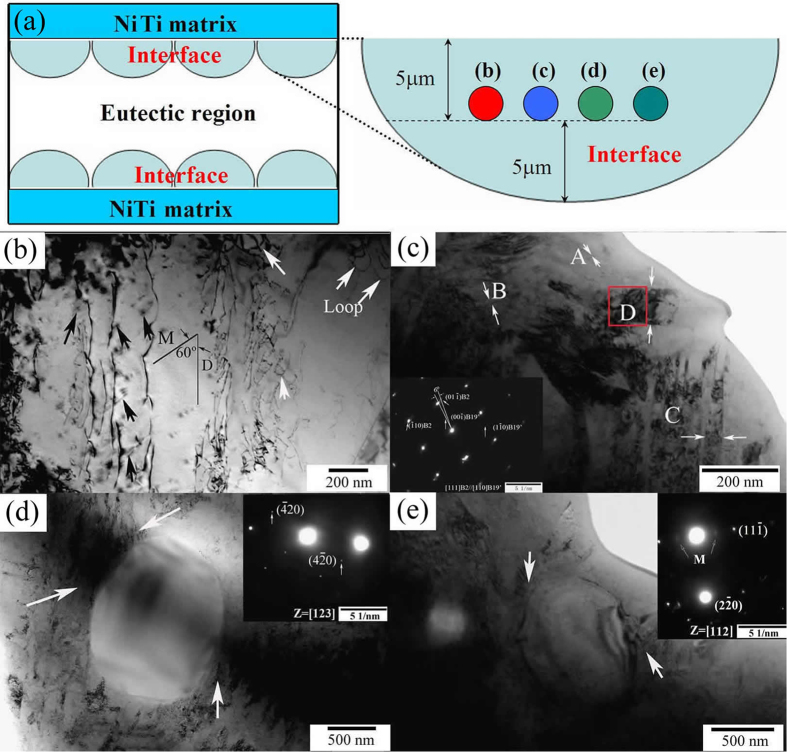
TEM micrographs in the interfaces of brazed region sintered at 1180 °C for 4 min: (**a**) schematic illustrations of the studied areas for the following images, (**b**) dislocations combined martensite nucleation, (**c**) coarse martensite with SAED pattern in region D (inset), (**d**) rounded Ti_2_Ni and (**e**) TiC particles surrounded by acicular martensite.

Figure 3 shows the interfaces of the brazed region sintered at 1180 °C for 4 min. Figure 3(a) schematically represents the locations from which the following TEM images were taken. B19′ martensites in NiTi alloy are shown as self-accommodated (001) compound twins and formed from B2 due to small energy barriers^32^,^33^,^34^,^35^. Therefore, all the images in the current work were taken from the middle interface of the brazed region. Martensite accompanied with dislocations at the interfaces of brazed NiTi-Nb region can be seen in Fig. 3(b). Moreover, parallel martensite plates with newly developed dislocation line segments and loops are presented in Fig. 3(b). Orientation relationship between martensite propagating (M) and dislocation line segments growth (D) is determined to be 60°. Figure 3(c) shows martensites in four different regions labeled by A, B, C, and D. The selected area electron diffraction (SAED) pattern in region D indicates that the orientation relationship between B2 matrix and B19′ matrix is [111]_B2_//[110]_B19′_ and (01-1)_B2_ deviates 6° from (001)_B19′_. Similar results were obtained in ref. 36. In addition, numerous martensite plates are observed and self-accommodated twins are coarsened. Nanometers sized martensites can be seen in region A. However, regions B and C display coarse wavy martensite of 10–20 nm and 40–50 nm respectively. In contrast, large martensite particles of 90–100 nm are observed in region D, which provides free space for growth. Figure 3(d,e) show Ti_2_Ni and TiC particles surrounded by acicular martensite. As seen from Fig. 3(d,e), acicular martensite prefers to separate out of around these particles. This is consistent with the fact that precipitations in NiTi alloy accelerate the B2 to martensite transformation.

**Figure 4:**
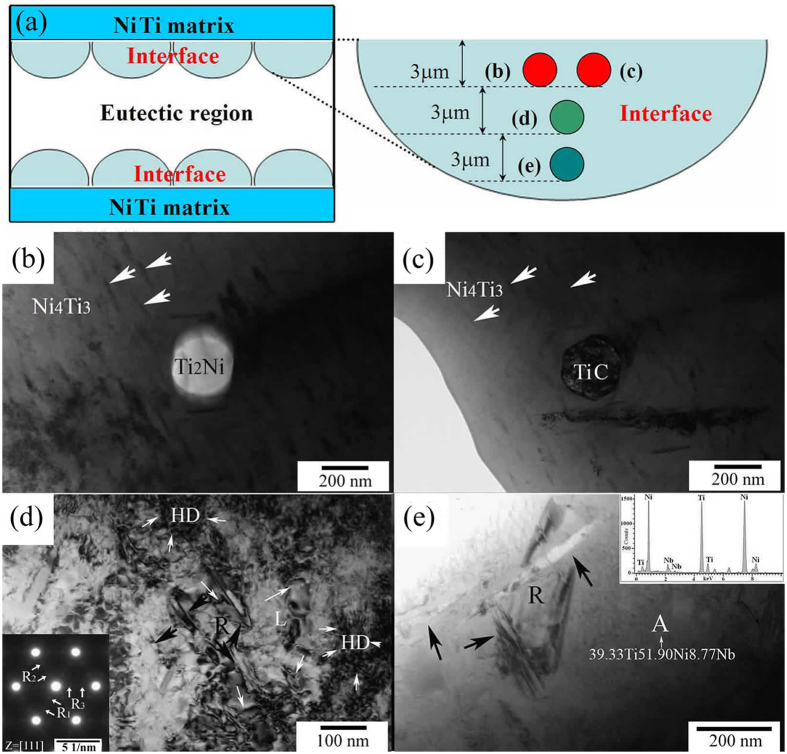
TEM micrographs of brazed region sintered at 1180 °C for 4 min followed by aged at 520 °C for 30 min: (**a**) schematic areas of the following TEM characterization, (**b**) Ni_4_Ti_3_ precipitates accompanied with rounded Ti_2_N particle, (**c**) Ni_4_Ti_3_ precipitates along with TiC particle, (**d**) R phase precipitates accompanying dislocation loops (L) and high density dislocation (HD) with a TEM SAED pattern of R phase region (inset), and (**e**) R phase present nearby eutectic region.

It is mentioned in^37^,^38^,^39^, the transformation behavior of superelasticity of nickel-rich NiTi alloy is sensitive to annealing temperature. Optimal compressive superelasticity was obtained after ageing at 520 °C due to Ni_4_Ti_3_ precipitation. These results could guide selecting suitable heat treatment parameters for preparing NiTi-Nb wires. TEM observations of the brazed region sintered at 1180 °C for 4 min followed by ageing at 520 °C are shown in Fig. 4. Figure 4(a) shows the studied areas for TEM observations. Similar to the round-shaped Ti_2_Ni and polygonal TiC particles shown in Fig. 3(d,e), Ti_2_Ni and TiC particles in Fig. 4(b,c) also separate out of the reaction interface in the aged specimen. Whereas no martensite as the characteristic of micro-twin can be detected around these particles and other phases are also not precipitated around either Ti_2_Ni or TiC particles. Thus, a clear interface marked by arrows is found. Figure 4(d) shows the dislocation loops (L) and high density dislocation (HD) around R phase and the corresponding SAED pattern (inset). As could be seen from Fig. 4(d), the dislocations and high density dislocation regions appear, and multi-variants of R phase can also be observed. In addition, R phase nucleates and grows freely surrounding the dislocations. Stress field caused by high density dislocation provides the driving force for R phase transformation, which accelerates the growth of R phase. Compared to B19′ martensite transformation, R phase transformation easily occurs because of its less nucleation energy^40^. This has been confirmed in the present study. Three different SAED patterns are distinguished for R phase (marked as R_1_, R_2_, and R_3_) in Fig. 4(d). Among the three different R phase variants, R_1_ variant easily separates out due to its lowest nucleation energy. On the other hand, R_3_ variant overcomes maximum obstacle to be precipitated, which is attributed to the inhomogeneous chemical composition (Ni, Ti, Nb) in R phase region in the unstable interface between NiTi matrix and eutectic. Longer R phase accompanied by the newly developed micro-twin R phase (black arrows) is visible at the interfaces nearby the eutectic region (Fig. 4(e)). The chemical composition in region A is measured to be 39Ti-52Ni-9Nb (wt%) (proeutectic phase). This region with unstable chemical composition benefits for the nucleation of R phase.

**Figure 5:**
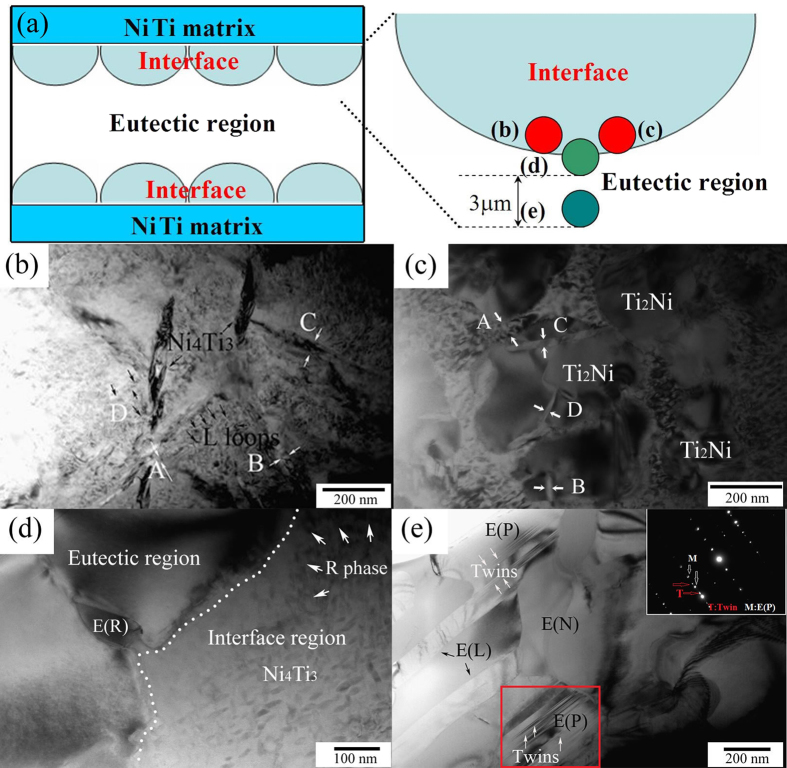
TEM micrographs of brazed region sintered at 1180 °C for 4 min followed by aged at 520 °C for 30 min: (**a**) schematic of the following TEM areas, (**b**) R phase combined Ni_4_Ti_3_ precipitates with dislocation loop and density dislocation region inside, as marked by the black arrows, (**c**) Ti_2_Ni particle accompanied with coarsening R phase precipitates, (**d**) Ni_4_Ti_3_ precipitates growing directly to the eutectic region combined R phase and rod-shaped eutectic (E(R)) present at the boundary between eutectic and interface region, and (**e**) details of phase composition in eutectic region: proeutectic NiTi(Nb) particles E(P) with twins inside, round-shaped Nb-rich phases E(N), and lamellar-shaped eutectic E(L).

R phase coupled with Ni_4_Ti_3_ precipitations and dislocation loop are observed in Fig. 5(b). Regions A, B, and C indicate the consecutive process nucleation, growth and coarsening of R phase. Largest width of R phase is observed in Region C. Furthermore, several nanometer-sized R phase freely separates out in region D. R phase transformation is mixed with dislocation loop at the interface of NiTi matrix and NiTi-Nb eutectic region. The amount of Ti_2_Ni gradually increases, thereby playing an important role in accelerating the precipitation of R phase, as clearly indicated in Fig. 5(c). A higher amount of R phase with different width is distributed in regions A, B, C, and D. The grain size of R phase decreases from the surface to the center of Ti_2_Ni, i.e. 84 nm in region A, 42 nm in region C, 35 nm in region B, and 23 nm in region D. This indicates that R phase preferably precipitates around the interface of Ti_2_Ni particles. From the aforementioned two martensite transformations, R-phase contributes to a very small transformation strain (about 1%), whilst B19′ phase causes a much larger transformation strain (around 10%) associated with a larger transformation barrier^41^. In addition, similar to refs 42 and 43, Ti_2_Ni particles promote the R phase transformation. The eutectic region was also investigated in details using TEM. Distinct boundary between eutectic region and interface region (marked by a series of black arrows) is observed in Fig. 5(d). Round-shaped Nb-rich phases E(N), lamellar-shaped eutectic E(L), and proeutectic NiTi(Nb) particles E(P) containing many twins can be found in Fig. 5(e) where no martensite precipitates was visible.

### Deformation behavior

**Figure 6:**
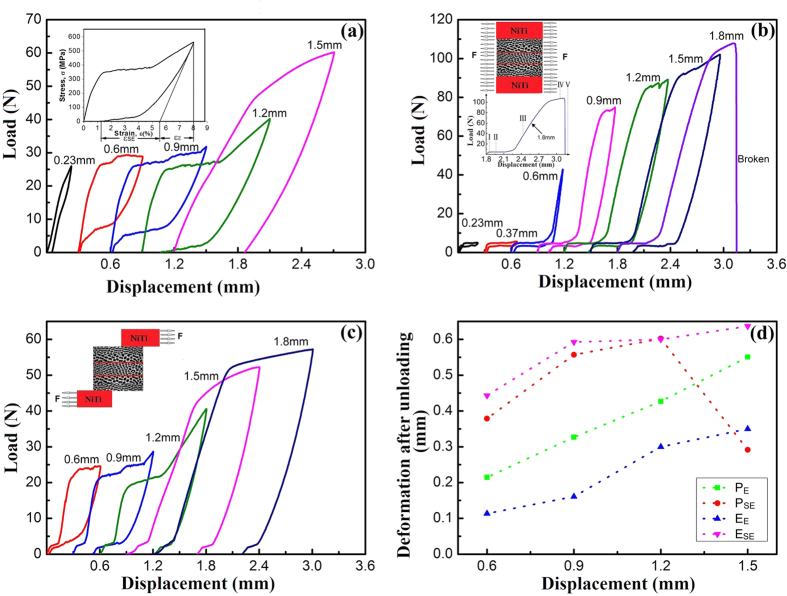
Cyclic loading–unloading tensile load-displacement curves of NiTi wire and brazed wires: (**a**) pure NiTi wires, (**b**) tensile specimen including only brazed area, (**c**) tensile specimen including both brazed area and pure NiTi wires, (**d**) comparison of strain after unloading for both pure NiTi wires (P) and brazed NiTi wires (E), as indicated in (**b**). P_SE_: Deformation values for superelastic recovery stage of pure NiTi wires; P_E_, Deformation values for elastic recovery stage of pure NiTi wires; E_SE_ and E_E_ are the values of brazed NiTi wires.

The cyclic loading–unloading tensile load-displacement curves of NiTi wire and brazed wires aged at 520 °C for 30 min are shown in Fig. 6. The superelastic characteristics of specimens could be described as *ε*_SE_ (superelastic strain) and *ε*_E_ (pure elastic strain) upon unloading, respectively, shown as inset in Fig. 6(a). Superior superelasticity is obtained for NiTi wire at the second and third cycles and the value of *ε*_R_ approaches zero. The load plateau during unloading cycle is becoming lower with increasing the tensile deformation. Consequently, a worst superelascity is obtained at the displacement of 1.5 mm (Fig. 6(a)). Distinct load-displacement curves for the brazed TiNi wires during mechanical cycling are obtained (Fig. 6(b,c)). As mentioned earlier, acicular-shaped martensite at the interface between two wires contributes to the deformation. Therefore, it can be concluded that, at the beginning of tensile deformation, R phase reorientation and R phase transformation occur in the apparently elastic region in the R phase state. It was reported that R phase transformation or reorientation strains are not constant, typical for common martensitic transformations^44^. From the two types of tensile specimens, a larger plastic deformation of R phase reorientation can be obtained for tensile specimen including only brazed area, whose cumulative deformation reaches up to 0.3 mm when the tensile displacement is in excess of 0.6 mm (Fig. 6(b)). Following the R phase reorientation and R phase transformation, plastic deformation of NiTi occurs followed by B19′ martensite transformation. It is mentioned that martensitic transformation occurs during various thermo-mechanical tests^44^. Inset in Fig. 6(b) shows the curve of the brazed NiTi wires deformed up to 1.8 mm, in which five distinct deformation stages, i.e. I–V, are found. Figure 6(d) describes the tensile displacement and deformation relationship after unloading for both pure NiTi wire (P) (Fig. 6(a)) and brazed NiTi wire (E) (Fig. 6(b)). The deformation values for both superelastic recovery stage (P_SE_, E_SE_) and elastic recovery stage (P_E_, E_E_) are clearly indicated in Fig. 6(d). It can be clearly observed that deformation value gradually increases after unloading with increasing the tensile displacement. However, deformation value during superelastic recovery stage for brazed NiTi wire (E_SE_) is found to be greater than that of pure NiTi wire (P_SE_) at the same tensile displacement. This can be attributed to the transformation from martensite to parent phase. During superelastic recovery stage for brazed NiTi wire, maximum deformation value is found to be about 0.67 mm at the tensile displacement of 1.5 mm. In addition, at the same tensile displacement, deformation value during elastic recovery stage for brazed NiTi wire is lower than that for pure NiTi wire.

### Deformation mechanisms

The loading-unloading curves in Fig. 6(b,c) exhibit five different stages (i.e. I, II, III, IV, and V) which indicate different deformation behaviors of the brazed NiTi wires. Although four different deformation behaviors have been reported in ref. 45, only three deformation stages (III, IV and V) are observed for NiTi wire during loading and unloading (Fig. 6(a)). Elastic deformation occurs during the first stage for the specimens in Fig. 6(b,c). This is associated with R phase reorientation. In addition, R phase transformation occurs from parent phase in stage II, where load follows a large load plateau for both specimens tested in the middle and out of brazed area, as shown in Fig. 6(b,c).

In the stage III, the elastic deformation of the reoriented martensite accompanied by the plastic deformation of Nb-rich phase and lamellar NiTi-Nb eutectic significantly contributed. Stage IV is related to the B19′ martensite transformation in NiTi matrix^46^. The final stage (stage V) mainly consists of plastic deformation process. According to literatures, R phase and B19′ martensite phase generally nucleate around Ni_4_Ti_3_ phase precipitates, inducing elastic strain field and the adjacent composition gradient^37^,^47^,^48^,^49^. Furthermore, B2 to R transformation easily takes place near the broad face of the Ni_4_Ti_3_ precipitates than B2 to B19′ transformation. This is attributed to the elastic interaction and solute inhomogeneity^50^. The Nb-rich phase also contributes to the elastic strain field, strong stress redistribution and surrounding composition gradient^49^. According to refs 51 and 52, Nb element with a larger atomic radius substituting the Ti sublattice in NiTiNb specimen contributes to higher energy dissipation during transformation. This helps to increase hysteresis. It is also mentioned, the martensite transformation temperature in NiTiNb alloy decreases with increasing Nb content and Ni/Ti ratio. This ultimately hinders martensite transformation^53^. Thus, R martensite phase is dominantly obtained at the interfaces between NiTi-Nb eutectic and NiTi wire at the low Nb content in the present work. During loading, a higher fraction of R phase transformation in the brazed NiTi wire also confirms that R phase transformation is readily found at the interface of eutectic microstructure and NiTi wire. This is also evidence showing that B2 to R phase transformation initially occurs, similar to ref. 50.

**Figure 7:**
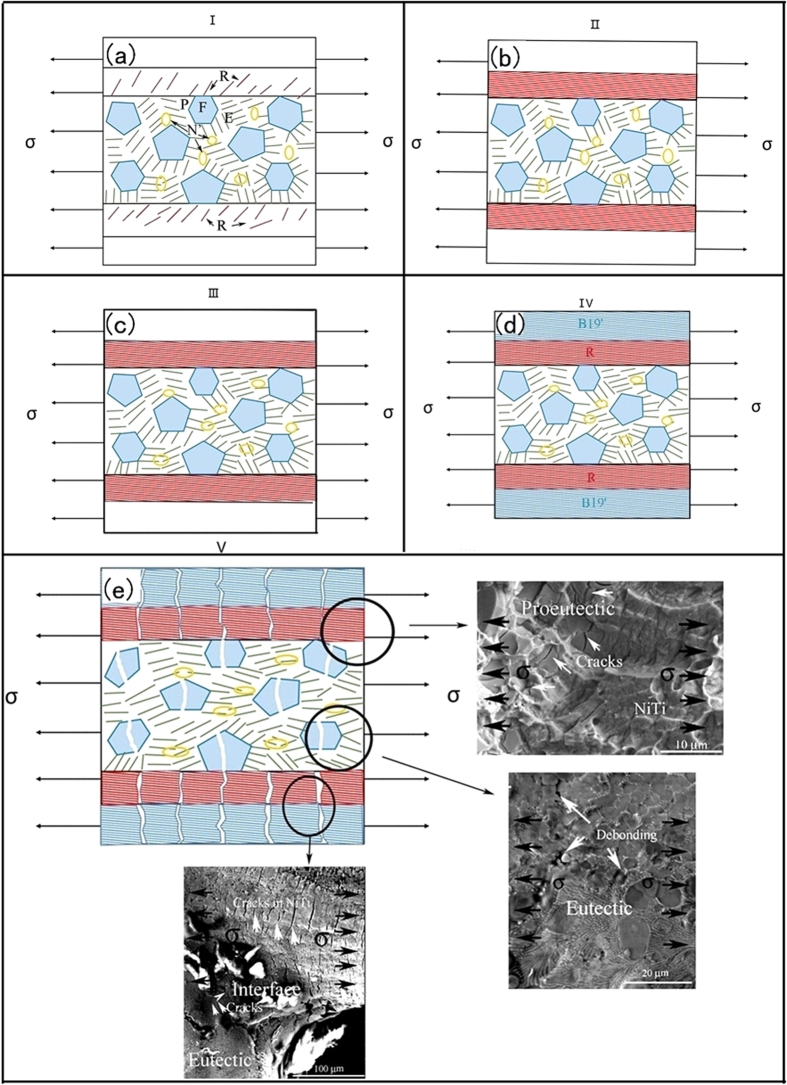
Schematic illustration of deformation behavior during loading processing for brazed NiTi wires followed by aged at 520 °C for 30 min: (**a**) Stage I, (**b**) Stage II, (**c**) Stage III, (**d**) Stage IV, and (**e**) Stage V.

As discussed above, five different deformation stages are obtained for brazed NiTi wires. This kind of deformation behavior during loading for brazed NiTi wires is schematically illustrated in Fig. 7. At the beginning of tensile deformation, R martensite reorientation to tensile direction takes place at the interfaces between eutectic microstructure and NiTi wire. A little change of other phases (P, E and N’) is observed as a result of small deformation in Stage I, as described in Fig. 7(a). At the second stage (Stage II, Fig. 7(b)), a higher fraction of R phase is induced, which significantly contributes to the elongation of the specimen. This has been showing as a platform in loading tensile curve in Fig. 6(b). During the third stage (Stage III), Nb-rich phase and lamellar NiTiNb eutectic align to the tensile direction (Fig. 7(c)). A small plastic deformation is obtained for Ti-rich particle owing to its higher hardness, which was previously discussed in ref. 26. With increasing tensile strain, B19′ martensite transformation occurs especially in NiTi matrix and also reorientates along the loading direction at Stage IV (Fig. 7(d)). This is accompanied by the deformation of Nb-rich phase and eutectic phase. Owing to the brittleness of Ti-rich particle (F), cracks are initially induced at these particles and propagate into the NiTi matrix. Meanwhile, a significant amount of cracks also generate in NiTi matrix as a result of its softness. Cracks propagation features at Stage V are shown in Fig. 7(e). Further evidence of crack propagation and eutectic phase extension of the distinct regions (A, B, and C) are observed in Fig. 7(e). In region A, many cracks generated and propagated after several cycles in NiTi matrix. It should be mentioned that cracks stretched perpendicular to tensile direction. The bonding strength in eutectic region is much stronger than that of NiTi wire. In contrast, no cracks can be found in eutectic area after several stress cycles in region B. Microstructure also stretches along the tensile direction. Further, soft Nb-rich phase also significantly contributes to the plastic deformation. In region C, many cleavage planes accompanied by few dimples can be found in the fracture morphologies of eutectic region. In addition, several cracks appear in the Ti-rich particles. This provides evidence of the brittle fracture of Ti-rich phase. In contrast, more ductile dimples are presented in the NiTi matrix side, which correlates well with the ductility of each phase.

## Conclusions

NiTi wires can be brazed together *via* liquid eutectic formation between NiTi and Nb powder deposited at the wire contact region. This is a pioneering work for producing superelastic NiTi scaffolds brazed from stacked, woven or braided wires. Phase transformation and deformation behavior of the NiTi-Nb eutectic microstructure, especially for the superelasticity, are investigated using TEM and cyclic loading-unloading tests. Following conclusions can be highlighted:

1. B2 to B19′ phase transformation is found to be retarded after heat treatment at 520 °C for 30 min for both NiTi wire and eutectic region. This confirms that a larger transformation barrier was present for B19′ phase transformation.
2. R phase transformation preferentially occurs at the interface between NiTi and eutectic region during solidification. Inconsistent Nb element accompanied by the presence of Nb-rich phase significantly contributes to the elastic strain field and surrounding composition gradient. Hence, a high fraction of R phase transformation is induced in this area.
3. Ti_2_Ni and TiC particles surrounded by acicular martensite are observed in the brazed NiTi after sintering, while no distinct martensite transformation can be obtained after aging at 520 °C for 30 min. However, a large amount of Ni_4_Ti_3_ particles was present.
4. Deformation mechanism of brazed NiTi wires is comprised of five different stages: (I) R phase reorientation, (II) R phase transformation from parent phase, (III) elastic deformation of reoriented martensite accompanied by plastic deformation of Nb-rich phase and lamellar NiTi-Nb eutectic, (IV) B19′ martensitic transformation as the deformation behavior for the NiTi matrix, and (V) plastic deformation of the specimen.

## Additional Information

**How to cite this article**: Wang, L. *et al.* Phase transformation and deformation behavior of NiTi-Nb eutectic joined NiTi wires. *Sci. Rep.* **6**, 23905; doi: 10.1038/srep23905 (2016).
